# A Design Method of Diffraction Structure Based on Metasurface for High-Resolution Spectroscopy

**DOI:** 10.3390/nano13182503

**Published:** 2023-09-05

**Authors:** Jingaowa Hu, Lingjie Wang, Shangnan Zhao, Haokun Ye

**Affiliations:** 1 Changchun Institute of Optics, Fine Mechanics and Physics, Chinese Academy of Sciences, Changchun 130033, China; hjgw0617@163.com (J.H.); wanglingjie@126.com (L.W.); yehaokun19@mails.ucas.ac.cn (H.Y.); 2 University of Chinese Academy of Sciences, Beijing 100039, China; 3 State Key Laboratory of Applied Optics, Changchun 130033, China; 4 Key Laboratory of Optical System Advanced Manufacturing Technology, Chinese Academy of Sciences, Changchun 130033, China; 5 Optoelectronic Information Engineering, School of Optoelectronic Engineering, Changchun University of Science and Technology, Changchun 130033, China

**Keywords:** metasurface, off-axis meta-lens, spectroscopy, multifunctional structure

## Abstract

In this paper, a design method of diffraction structure based on metasurface is proposed for light splitting and focusing simultaneously. In the method, firstly, the light field calculation model of the proposed structure is established based on Fresnel diffraction and the transmittance function is calculated. Then, the model structural parameter selection mechanism is determined, and the spectrum resolution equation of the structure is derived. Simulation results indicate that the proposed method can offer a broader working bandwidth and enhanced higher resolution compared to off-axis meta-lens. Moreover, this proposed method can be deployed in high-resolution, wide-band ultra-compact spectrometer systems potentially.

## 1. Introduction

Recently, as research on metamaterials and metasurface progresses tirelessly, the prospect of dispersion modulation through the implementation of these materials has opened up. Meta-lens is a planar optical device composed of two-dimensional metamaterial that can manipulate the phase, polarization, amplitude, and other properties of light flexibly [1]. According to the applications, different types of meta-lens have been designed, such as achromatic meta-lens [2,3,4,5,6], meta-lens-based optical polarization sensor [7], meta-lens-based bandpass filters [8], sub-resolution meta-lens [9,10], color holography [11,12,13], multifunctional meta-lens [14,15,16,17], adaptive meta-lens [18,19,20], integrated photonics [21,22,23] and spectrometers [24,25,26].

The spectrometer is a valuable tool for measuring spectra. It has been applied in many fields, such as space surveillance, airborne and spaceborne remote sensing, geologic analysis, environmental monitoring, medical detection and diagnosis, and military detection [27]. Conventional spectrometers typically consist of isolated dispersive elements and focusing lenses, resulting in bulky and non-portable instruments, generally utilized for research inside laboratories and specific applications. Due to the swift advancement of diverse areas, the size and weight of analytical instruments have become more demanding. Thus, spectrometers’ trend has shifted towards miniaturization, intelligence, and portability. Advances in micro and nanofabrication technologies have provided opportunities to reduce the size of systems to achieve micro-spectrometers [28], and based on this direction, researchers have begun to explore the potential of using the properties of metasurface to achieve micro-spectrometers. Compared to diffractive components, such as Fresnel lenses, meta-lens avoid the fundamental limitation of multiple diffraction orders [29], and the planar structural features of meta-lens offer the advantages of simple fabrication, low insertion loss, and the ability to modulate the spatial distribution of reflected and transmitted optical fields in a compact form [30], opening a novel avenue to the design and preparation of ultra-compact spectrometers for high-resolution, broadband spectroscopy.

In 2016, Khorasaninejad et al. investigated a compact spectrometer in the near-infrared band based on an off-axis meta-lens with an off-axis beam angle of 80°, the ability to resolve wavelength differences as small as 200 pm, and a focusing efficiency of around 30%. Furthermore, the operating bandwidth of the spectrometer is expanded by stitching multiple off-axis meta-lens [24]. In 2019, Capasso et al. implemented an ultra-compact spectrometer operating in the visible wavelength band using off-axis meta-lens, likewise by stitching multiple off-axis meta-lens to broaden the operating bandwidth [25,26]. The current method of stitching together structures to obtain a wide operating band increases the structural area and poses some difficulties in processing and mounting. Therefore, a better design of the structure with light splitting and focusing needs to be further investigated for high-resolution ultra-compact spectrometers to obtain a smaller system size and a wider working band.

In this paper, a design method of diffraction structure based on metasurface is proposed for light splitting and focusing simultaneously. Firstly, the light field calculation model is established based on Fresnel diffraction, and the transmittance function of the diffraction structure is calculated by the phase distribution of the designed structure. On this basis, the rules for the selection of the structure parameters are elaborated, and the resolution equation of the structure is derived. Then, in order to verify the feasibility of the structure design method, a diffraction structure with a diameter of 60 μm and an off-axis angle of 10°, designed for the wavelength of 1550 nm, is simulated. Simulation results indicate that the proposed structure has higher resolution, wider working band, and better imaging quality compared to off-axis meta-lens with the same parameters.

## 2. Structures and Methods

There are three types of metasurface to achieve phase modulation in the 0~2π range: transport-phase type, resonant-phase type and Pancharatnam–Berry phase type [31]. In this paper, a transport-phase type metasurface is used to achieve phase coverage from 0 to 2π by selecting a cell structure with various structural parameters.

**Figure 1:**
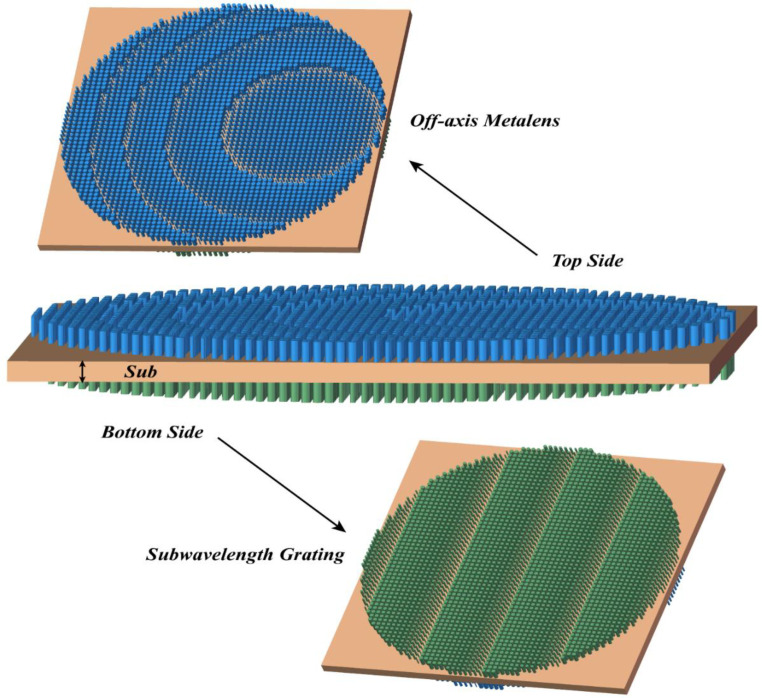
Schematic diagram of the double-layer diffraction structure.

In this paper, a diffraction structure design method based on metasurface is proposed. Utilizing the method, a bilayer model is designed in this part; the top surface is an off-axis meta-lens used to achieve focusing and partial light splitting, the bottom surface is a linear subwavelength grating used to achieve light splitting by designing the phase distribution, the double-layer diffraction structure is as shown in Figure 1.

**Figure 2:**
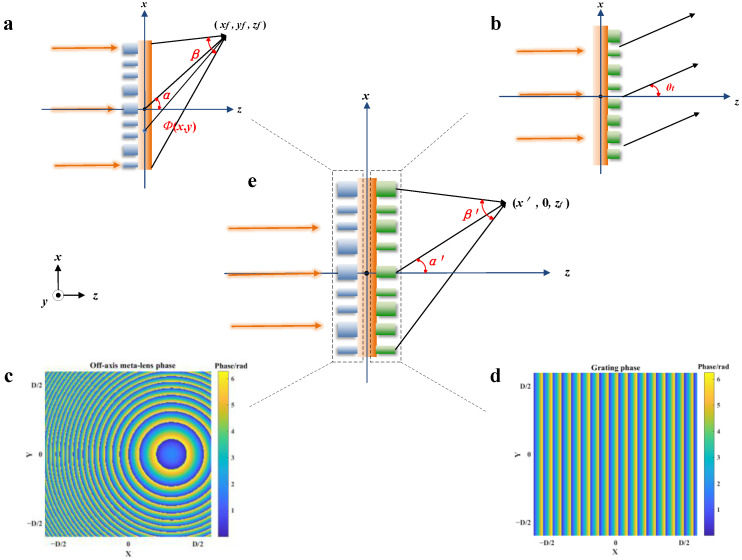
Schematic of the proposed structure. (**a**) Focusing on an off-axis meta-lens, (**b**) deflecting of the subwavelength grating, (**c**) Off-axis meta-lens phase distribution, (**d**) Subwavelength grating phase distribution, and (**e**) Focusing on the proposed structure.

Aiming at the proposed diffraction structure, the light field calculation model is established, and the transmittance function of the double-layer diffraction structure is calculated by the phase distribution of the top and bottom surfaces of the designed structure. The schematic of the proposed structure is shown in Figure 2, where α is the off-axis angle of the off-axis meta-lens, *β* is the focusing angle of the off-axis meta-lens, *θ_t_* is the deflection angle of grating to the incident beam, *α*′ is the overall off-axis angle of the proposed structure, *β*′ is the overall focusing angle of the proposed structure, and *D* is the diameter of the proposed structure.

As shown in Figure 2a, with the off-axis meta-lens focus outside the optical axis, the phase distribution of the off-axis meta-lens can be calculated according to Equation (1) for the selected focus position (*x_f_*, *y_f_*, *z_f_*).

(1) $$\Phi (x,y)=\frac{2\pi }{\lambda }\left[f-\sqrt{{\left(x-x_{f}\right)}^{2}+{\left(y-y_{f}\right)}^{2}+z_{f}{}^{2}}\right]$$

The transmittance function for an off-axis meta-lens can be calculated from Equation (1) as:

(2) $$g(x,y)=\exp\left[i\Phi (x,y)\right]=\exp\left[i\frac{2\pi }{\lambda_{d}}\left(f-\sqrt{{\left(x-x_{f}\right)}^{2}+{\left(y-y_{f}\right)}^{2}+z_{f}{}^{2}}\right)\right]$$

where *λ_d_* is the design wavelength, $f=\sqrt{x_{f}^{2}+y_{f}^{2}+z_{f}^{2}}$.

According to the light splitting and focusing properties of off-axis meta-lens, (*x* − *x_f_*)^2^ + (*y − y_f_*)^2^ << *z_f_*^2^, so that Equation (2) can be simplified to the following equation:

(3) $$\begin{array}{ll} \sqrt{{\left(x-x_{f}\right)}^{2}+{\left(y-y_{f}\right)}^{{}^{2}}+z_{f}{}^{2}} & =z_{f}\sqrt{1+\frac{{\left(x-x_{f}\right)}^{2}+{\left(y-y_{f}\right)}^{2}}{z_{f}^{2}}}\\ & »z_{f}+\frac{{\left(x-x_{f}\right)}^{2}+{\left(y-y_{f}\right)}^{2}}{2z_{f}} \end{array}$$

Combining Equations (2) and (3), the transmittance function for an off-axis meta-lens is obtained as:

(4) $$g(x,y)=\exp[i\frac{2\pi }{\lambda_{d}}(f-z_{f}-\frac{{\left(x-x_{f}\right)}^{2}+{\left(y-y_{f}\right)}^{2}}{2z_{f}})]$$

The subwavelength grating located on the bottom surface selects a linear phase distribution using the phase distribution function presented below:

(5) φ(x)=2πA⋅x+πAD

where *A* is the phase slope of the linear grating, *D* is the diameter of the proposed structure, and *x* is the coordinate position of the cell distribution.

According to Equation (5), the transmittance function of the subwavelength grating can be obtained as:

(6) $$\begin{array}{ll} h(x) & =\exp[i\varphi (x)]\\ & =\exp[i(2\pi Ax+\pi AD)] \end{array}$$

By combining the off-axis meta-lens with a one-dimensional grating on the same substrate, the complex amplitude transmittance function is:

(7) u(x,y)=h(x)g(x,y)

Placing the combined structure in the *z* = 0 plane and illuminating it with a plane light wave, the complex amplitude *U*(*x*′, *y*′) of the diffracted light in any plane at *z* > 0 can be obtained from the Fresnel integral:

(8) $$U(x^{\prime },y^{\prime })=\iint u(x,y)\exp\left\{\frac{i\pi }{\lambda z}\left[{\left(x-x^{\prime }\right)}^{2}+{\left(y-y^{\prime }\right)}^{2}\right]\right\}dxdy$$

Taking Equations (4), (6) and (7) into Equation (8) and simplifying them, combining the relevant terms gives:

(9) $$U(x^{\prime },y^{\prime })=C\cdot \iint \exp\left[i2\pi \left(x^{2}+y^{2}\right)\left(-\frac{1}{2\lambda_{d}z_{f}}+\frac{1}{2\lambda z}\right)\right]\cdot \exp\left[i2\pi \left(Ax+\frac{xx_{f}+yy_{f}}{\lambda_{d}z_{f}}-\frac{xx^{\prime }+yy^{\prime }}{\lambda z}\right)\right]dxdy$$

In Equation (9), *C* is a constant indicating strength, $C=\exp\left[i2\pi \left(\frac{f}{\lambda_{d}}-\frac{z_{f}}{\lambda_{d}}-\frac{x_{f}^{2}+y_{f}^{2}}{2\lambda_{d}z_{f}}+\frac{{x^{\prime }}^{2}+{y^{\prime }}^{2}}{2\lambda z}\right)+i\pi AD\right]$.

In this paper, the off-axis meta-lens *y_f_* = 0 on the top surface of the discussed double-layer structure, as shown in Figure 2e. According to Equation (9), in the *x*′-*z* plane, the spectral brightest point of wavelength *λ* appears when the following conditions are satisfied:

(10) $$\begin{array}{l} x^{\prime }=x_{f}+\lambda_{d}z_{f}A\\ y^{\prime }=0\\ z=\frac{\lambda_{d}}{\lambda }z_{f} \end{array}$$

Meanwhile, to ensure the diffraction efficiency of the designed structure, the grating has to deflect the beam at the same angle as the focus angle of the off-axis meta-lens. Therefore, after determining the off-axis angle *α* of the off-axis meta-lens, the deflection angle *θ_t_* (as shown in Figure 2b) of the grating is also determined. Furthermore, the slope of the phase distribution *A* of the subwavelength grating structure can be deduced from the generalized Sneer’s law, which is derived as follows:

(11) $$n_{t}\sin\theta_{t}-n_{i}\sin\theta_{i}=\frac{\lambda }{2\pi }\cdot \frac{d\varphi }{dx}$$

In this paper, parallel light is incident perpendicular to the structure (*θ_i_* = 0), and the transmitted space is air (*n_t_* = 1), taking Equation (5) into Equation (11) will obtain:

(12) $$\begin{array}{ll} \sin\theta_{t} & =\frac{\lambda }{2\pi }\cdot \frac{d\varphi }{dx}\\ & =\lambda \cdot A \end{array}$$

Thus, the slope of the phase distribution *A* in the subwavelength linear grating can be computed based on the deflection angle *θ_t_* of the grating to the incident beam, thereby facilitating the determination of the subwavelength grating structures.

There are three major factors that determine the spectral resolution of meta-lens: focal length, off-axis angle and numerical aperture. The minimum discriminable wavelength difference in the focal plane (*δλ*_min_) can be determined according to the Rayleigh criterion ($\frac{0.61\lambda }{NA}$), as follows [24]:

(13) $$\begin{array}{ll} \delta \lambda_{\min} & =\frac{d\lambda }{\left|\Delta \vec{r}\right|}\cdot \frac{0.61\lambda_{d}}{NA}\\ & =\frac{d\lambda }{f\{\sin^{-1}[(1+\frac{d\lambda }{\lambda_{d}})\sin\alpha ]-\alpha \}}\cdot \frac{0.61\lambda_{d}}{\sin(\frac{1}{2}\tan^{-1}\frac{4F\cos\alpha }{4F^{2}-1})} \end{array}$$

where $f=\sqrt{x_{f}^{2}+z_{f}^{2}}$ is the focal length of off-axis meta-lens, $\left|∆\overset{⃑}{r}\right|$ is the distance displaced from the focus position due to changing the incident wavelength, *F* is the F-number (*F* = *f*/*D*) of the off-axis meta-lens, *λ_d_* is the design wavelength, d*λ* is the deviation of the design wavelength from the working wavelength, $NA=sin(\frac{1}{\beta })$ can be determined by the law of cosines, and *α* is the off-axis angle of the off-axis meta-lens.

Similarly, the resolution of the proposed structure is calculated as:

(14) $$\begin{array}{ll} \delta \lambda_{\min} & =\frac{d\lambda }{\left|\Delta \vec{r}\right|}\cdot \frac{0.61\lambda_{d}}{NA}\\ & =\frac{d\lambda }{f^{\prime }\{\sin^{-1}[(1+\frac{d\lambda }{\lambda_{d}})\sin\alpha^{\prime }]-\alpha^{\prime }\}}\cdot \frac{0.61\lambda_{d}}{\sin(\frac{1}{2}\cos^{-1}(\frac{2{x^{\prime }}^{2}+2z_{f}^{2}-\frac{D^{2}}{2}}{2\sqrt{{\left(x^{\prime }-\frac{D}{2}\right)}^{2}+z_{f}^{2}}\cdot \sqrt{{\left(x^{\prime }+\frac{D}{2}\right)}^{2}+z_{f}^{2}}}))} \end{array}$$

where $\left|∆\overset{⃑}{r}\right|$ is the displacement distance of the focus position due to changing the incident wavelength, *λ_d_* is the design wavelength, d*λ* is the deviation of the design wavelength from the working wavelength, $NA=sin(\frac{1}{2}\beta^{\prime })$ can be determined by the law of cosine, $f^{\prime }=\sqrt{x^{\prime 2}+z_{f}^{2}}$ is the overall focal length of the proposed structure, $\alpha^{\prime }=\tan^{-1}\left(\frac{x^{\prime }}{z}\right)$ is the angle of the overall off-axis focus of the proposed structure, *x*′ and *z_f_* are the *x* and *z* coordinates of the focus position of the proposed structure, respectively, and *D* is the diameter of the proposed structure.

Based on the above theoretical model, a double-layer structure based on metasurface is designed in this paper, with the parameters shown in Table 1.

**Figure 3:**
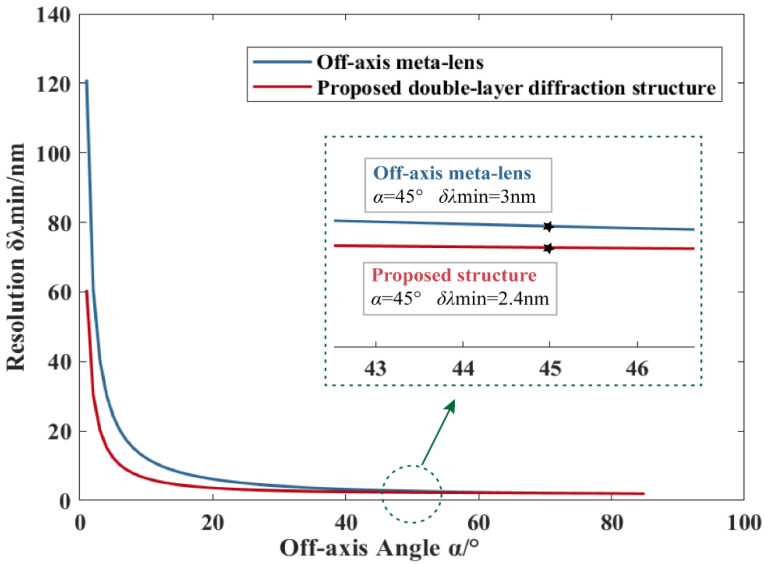
Comparison of the resolution of off-axis meta-lens and the proposed structures.

With the parameters in Table 1, the resolution of off-axis meta-lens is 3 nm [24], and the resolution of the proposed structure is raised to 2.4 nm, which can be calculated from Equation (14). In addition, since the dispersion capability of the off-axis meta-lens is positively correlated with the off-axis angle, the resolution of the proposed structure and the off-axis meta-lens have been compared by changing only the off-axis angle based on the above parameters and the comparison results are shown in Figure 3.

When plotting the resolution curves corresponding to the proposed structure in Figure 3, the slope of the phase distribution *A* for different off-axis angles is calculated from Equation (12). As shown in Figure 3, compared to the off-axis meta-lens, the proposed structure exhibits higher resolution at various off-axis angles.

## 3. Simulation Results and Discussion

In order to verify the correctness of the whole design process and the derivation of the equations, we carried out modeling and simulation in Ansys Lumerical 2023 R1.3, with linearly polarized light parallel to the *x*-axis as the light source, perfectly matched layer (PML) as a boundary condition, and the default adaptive mesh for simulation experiments. The structure used in the simulation verification process is a double-layer diffraction structure with a diameter of 60 μm and a design wavelength of 1550 nm, which is designed based on the selected structural parameters suitable for the device simulation, as shown in Table 2.

To avoid the effects of polarization, the unit structure is chosen as a cylindrical structure with a height of 1 μm and a period of 0.5 μm. The unit structure is composed of a nanopillar with Si and a substrate with SiO_2_; the radius distribution of the nanopillars on either side of SiO_2_ is determined by the calculated phase from the top and bottom sides.

**Figure 4:**
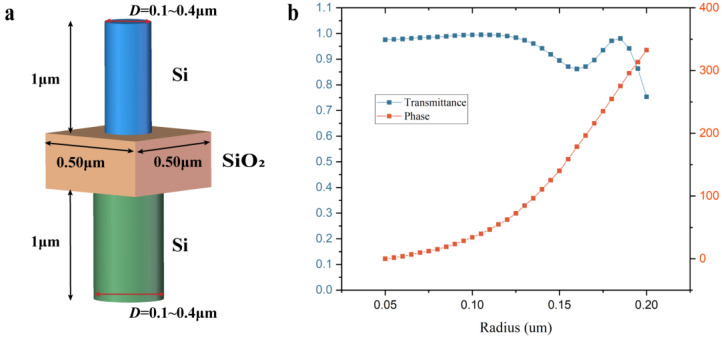
(**a**) Schematic diagram of the unit structure; (**b**) Phase and transmittance corresponding to different radius unit structures.

The unit structures of different radii are scanned to obtain the corresponding phases, and those covering the phase range from 0 to 2π are selected to build up a phase library at that parameter. As the nanopillars on both sides of SiO_2_ utilize the identical phase library, their diameter range is also the same, ranging from 0.10 μm to 0.4 μm, as shown in Figure 4a. It is important to note that the double-layer structure appears to have a common substrate, but it is actually a stack of two unit structure substrates, and the unit structure used to build the phase library is a combination of substrate and nanopillar. Therefore, the phase library built is the result of different radii of the nanopillar and substrate working together to modulate light. The phase and transmittance corresponding to different radius unit structures are shown in Figure 4b. During simulation experiments of unit structures, the boundary condition in the X and Y directions is Periodic, while in the *z* direction, PML is used.

According to the parameters in Table 2, the phase distribution is calculated by combining Equations (1) and (5), and the unit structure with a phase that closely matches the theoretical calculation is selected from the pre-established phase library.

**Figure 5:**
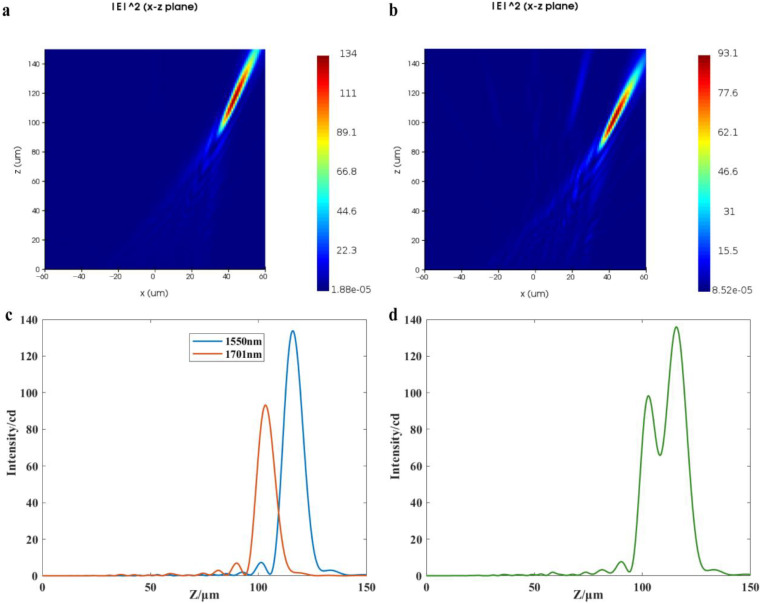
(**a**) Simulated light field in the *x*-*z* plane of the proposed structure with the incident wavelength of 1550 nm; (**b**) Simulated light field in the *x*-*z* plane of the proposed structure with the incident wavelength of 1701 nm; (**c**) Light intensity distribution in the *z* direction at *x* = 43 μm for *λ_d_* and *λ* incidence; (**d**) Superposition of the two light intensity curves in (**c**).

The simulation results are shown in Figure 5. After diffraction of parallel light with a wavelength of *λ_d_* = 1550 nm through the proposed structure, the simulation has determined that the focal point is located at (43 μm, 0, 116 μm), as shown in Figure 5a. According to Equation (14), the resolution of the double-layer diffraction structure at this parameter is calculated to be 150.1 nm, so the wavelength interval of 151 nm is chosen for the verification of resolution, that is, *λ* = 1701 nm. When the wavelength of the incident light is 1701 nm, the focus position obtained from the simulation is (42 μm, 0, 102 μm), as shown in Figure 5b. To facilitate the measurement, the dispersion of the off-axis meta-lens in the *x*-direction is neglected, and the monitor is placed at *x* = 43 μm. The light intensity curves in the *z*-direction at *λ_d_* and *λ* incidence are observed, as shown in Figure 5c. The two light intensity curves are superimposed, and the middle minima of the superimposed light intensity curves are 65.85 cd, and the minimum maximum value is 98.04 cd, as shown in Figure 5d. Based on Rayleigh’s criterion, the intermediate minimum is 81% smaller than the maximum peak, indicating that the proposed structure can resolve the incident light at 1550 nm and 1701 nm. Therefore, the spectral resolution predicted by the simulation corresponds with the theoretical calculations, and the deviation is less than 1%.

**Figure 6:**
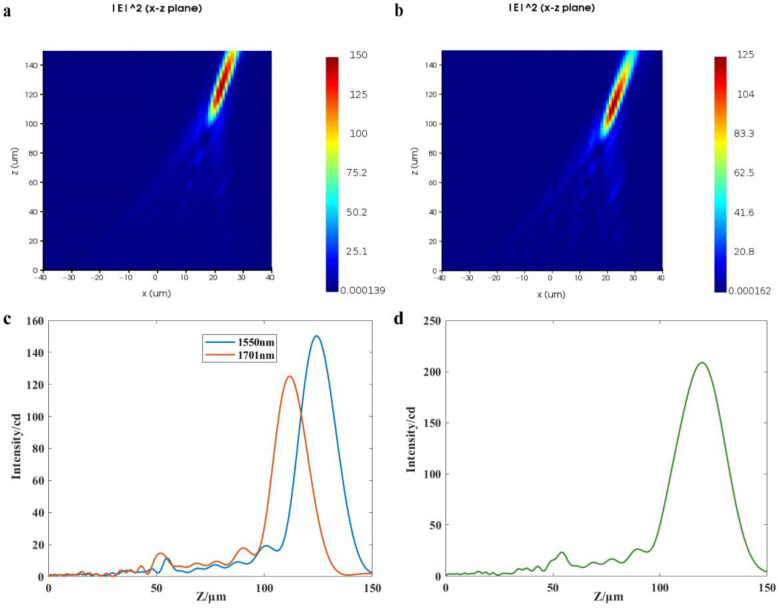
(**a**) Simulated light field in the *x*-*z* plane of the off-axis meta-lens at an incident wavelength of 1550 nm; (**b**) Simulated light field in the *x*-*z* plane of the off-axis meta-lens at an incident wavelength of 1701 nm; (**c**) Light intensity distribution in the *z* direction at *x* = 22 μm for *λ_d_* and *λ* incidence; (**d**) Superposition of the two light intensity curves in (**c**).

Then, the proposed structure and the off-axis meta-lens are simulated and analyzed using identical parameters to ensure consistency and accuracy in the resulting resolution. According to Equation (13), the resolution of the off-axis meta-lens with the parameters in Table 2 is 287.4 nm. With the wavelength *λ_d_* = 1550 nm and *λ* = 1701 nm, the resolution of the proposed structure and the off-axis meta-lens are compared. Simulation results indicate that the focus position obtained by the simulation of the off-axis meta-lens is (22 μm, 0, 124 μm) when the parallel light with the wavelength of 1550 nm is passed through the off-axis meta-lens, as shown in Figure 6a. When the incident wavelength is 1701 nm, the off-axis meta-lens simulation has determined that the focal point is located at (22 μm, 0, 112 μm), as shown in Figure 6b. The monitor is placed at *x* = 22 μm, and the light intensity curve in the *z*-direction at *λ_d_*, *λ* incidence is observed, as shown in Figure 6c. By superimposing the two light intensity curves, it can be seen that the off-axis meta-lens are unable to resolve the incident light at 1550 nm and 1701 nm, obviously, as shown in Figure 6d, which is consistent with the expected results.

**Figure 7:**
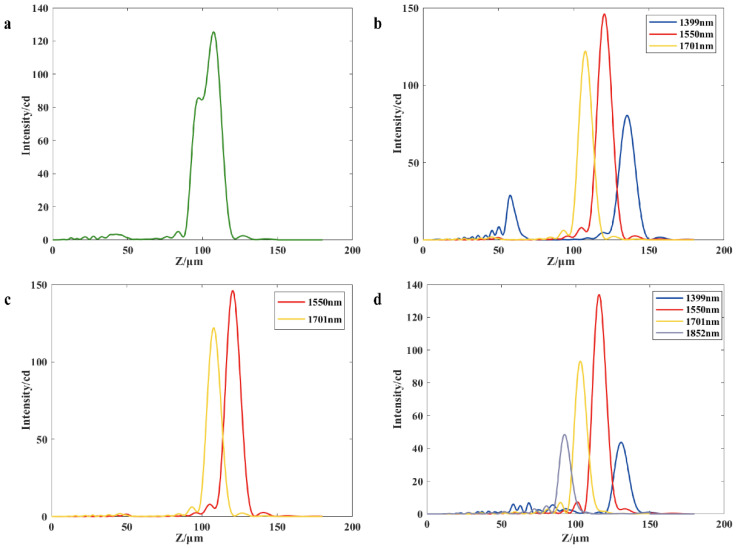
(**a**) Summation of the light intensity profiles for beams operating at 1701 nm and 1852 nm through an off-axis meta-lens; (**b**) Operating bandwidth distribution of an off-axis meta-lens; (**c**) For an off-axis meta-lens, there is crosstalk in the light intensity distribution at 1399 nm (**b**), so this band has been removed; (**d**) Operating bandwidth distribution of the proposed structure.

Next, the operating bandwidth of operation between the proposed structure and off-axis meta-lens with the same parameters is simulated and analyzed. Based on the above simulation experiments, it can be seen that the resolution of the proposed structure with *D* = 60 μm, *f* = 137.08 μm and *α* = 10° is 150.1 nm. The off-axis angle of off-axis meta-lens with the same resolution is 19.32° at the same structure diameter and focal length. The off-axis meta-lens and the proposed structure are simulated for the wavelength range of 1399 nm to 1852 nm, using 151 nm as the wavelength interval. Figure 7a–c shows the simulation results of the operating bandwidth of the off-axis meta-lens, and Figure 7d shows the simulation results of the proposed structure. The results indicate that the off-axis meta-lens are unable to distinguish between the operating wavelengths of 1701 nm and 1852 nm (Figure 7a). Additionally, the corresponding light intensity distribution is more crosstalk when the operating wavelength is 1399 nm (Figure 7b), which will produce a misjudgment phenomenon for other operating wavelengths. Therefore, 1399 nm is shaved off from the operating bandwidth; that is, the operating bandwidth of the off-axis meta-lens is 1550~1701 nm, as shown in Figure 7c. However, the crosstalk of the light intensity distribution within the 1399~1852 nm bandwidth of the proposed structure is small and distinguishable. Thus, the working bandwidth of the proposed structure is 1399~1852 nm, as shown in Figure 7d, which is almost twice the working bandwidth of the off-axis meta-lens.

Finally, the imaging quality of the proposed structure with off-axis meta-lens at the same parameters is analyzed using the Strehl Ratio (SR) for the evaluation of the imaging quality of the metasurface. When the SR is above 0.8, the imaging quality of the system is considered to be not significantly different from the ideal system [32]. The calculation equation is as follows:

(15) $$\mathrm{Strehl ratio}=\frac{I_{real}}{I_{ideal}}={\left|\iint e^{i\psi (x,y)}dxdy\right|}^{2}$$

where *I_real_* is the light intensity at the center of the actual point image formed by the proposed structure, *I_ideal_* is the central light intensity corresponding to the point diffusion function under ideal aberration-free conditions, and *Ψ*(*x*, *y*) is the wavefront difference between the actual wavefront and the ideal wavefront, with the integration range being the entire structure surface.

**Figure 8:**
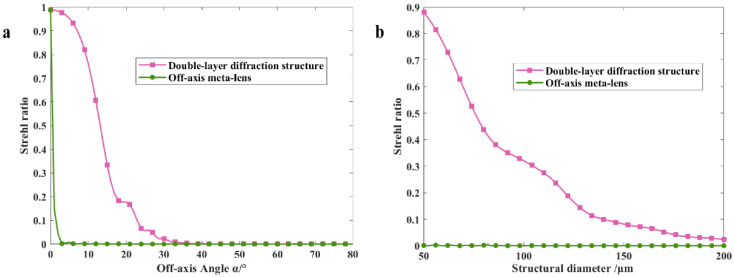
(**a**) SR curves for the proposed structure and off-axis meta-lens obtained by changing only the off-axis angle *α* with all other parameters unchanged; (**b**) SR curves for the proposed structure and the off-axis meta-lens obtained by changing only the structural diameter *D* with all other parameters unchanged.

Based on the simulation parameters in Table 2, the SR of the proposed structure and the off-axis meta-lens with the same parameters are evaluated. The SR of the proposed structure is 0.76, while the SR of the off-axis meta-lens is 3.1 × 10^−5^, indicating superior imaging quality for the former. In addition, to analyze the effect of off-axis angle and diameter on the imaging quality of the proposed structure, SR curves are calculated for the proposed structure with different off-axis angles and diameters, which have been compared to SR curves of off-axis meta-lens with the same parameters, as shown in Figure 8a,b. The results indicate that SR curves of both the proposed structure and the off-axis meta-lens show a rapid decrease with increasing off-axis angle and structure diameter; that is, the imaging quality becomes worse, but the SR of the proposed structure is always higher than the off-axis meta-lens. Furthermore, according to Equations (13) and (14), the spectral resolution is enhanced as the off-axis angle and structure diameter increase. Therefore, a reasonable choice of parameters is essential for achieving a harmonious balance between imaging quality and resolution.

In summary, compared to the off-axis meta-lens, the double-layer diffraction structure can provide higher resolution and superior imaging quality at the same off-axis angle. Furthermore, it can offer wider operating bandwidth and superior imaging quality with the same resolving ability, supplying a novel approach for the design of broadband, high-resolution ultra-compact spectrometer, as shown in Table 3.

Currently, processing methods for meta-lens include direct-write lithography, pattern transfer lithography, and hybrid patterning lithography [33]. As the laser direct-write lithography utilizes a computer-controlled optical system to project the desired nanopatterns directly onto the photoresist without the use of a mask, it offers better flexibility and higher accuracy than other processing techniques and is more suitable for processing the diffractive structure proposed in this paper. It should be noted that as the proposed structure is double-layer, proper alignment between the top and bottom layers should be ensured during the processing stage. Therefore, simplification of the structure is considered to achieve a single-layer structure with the same function.

The integral transmittance function of the proposed structure is derived from the direct multiplication of the transmittance functions of the top and bottom structures. It is known that the transmittance function of top and bottom structures is exponential function, so by expanding Equation (7), we can obtain:

(16) $$\begin{array}{ll} u(x,y) & =h(x)g(x,y)\\ & =\exp[i\varphi (x)]\exp[i\Phi (x,y)]\\ & =\exp[i\Phi^{\prime }(x,y)] \end{array}$$

where Φ′(*x*, *y*) is a new phase distribution of the single-layer structure.

**Figure 9:**
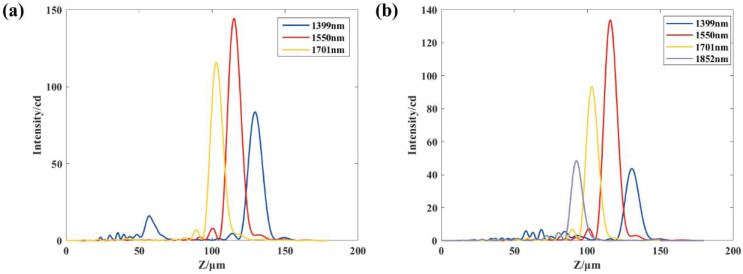
(**a**) Operating bandwidth distribution of the single-layer structure; (**b**) Operating bandwidth distribution of the double-layer structure.

Therefore, the new phase distribution Φ′(*x*, *y*) is considered to be used for modeling the simulation of the single-layer structure to achieve the simplification of the structure. For modeling a single-layer structure, the same structural parameters (as shown in Table 2), phase libraries, and simulation settings are used for a double-layer diffraction structure. To compare the operating bandwidth of double-layer and single-layer structures, the single-layer structure is also simulated for the wavelength range of 1399 nm to 1852 nm, using 151 nm as the wavelength interval. Throughout the verification process, each adjacent pair of wavelengths is judged using the Rayleigh criterion, and the wavelengths that satisfy the Rayleigh criterion are shown in Figure 9. As shown in Figure 9a, the single-layer structure obtained by the integral transmittance function has some crosstalk in the distribution of the *z*-direction at the wavelength of 1399 nm similar to the off-axis meta-lens, but the crosstalk is smaller, so the wavelength is still considered in the range of the operating bandwidth. Away from the design wavelength of the working wavelength, beam energy will gradually reduce, and in a double-layer structure, due to two layers of energy loss, light energy attenuation will be faster, so the minimum value of the summed light intensity of adjacent wavelengths is more likely to be lower than the minimum, maximum value of 81%. Therefore, compared with the single-layer structure obtained from the overall transmittance function, the double-layer diffractive structure will have a broader working band, as shown in Figure 9b.

In summary, the single-layer structure obtained by the overall transmittance function has the same resolving power as the double-layer structure, but the working bandwidth will be reduced. However, the double-layer diffractive structure might encounter difficulties in processing alignment. The choice of the corresponding structure can be made based on the needs of the application.

## 4. Conclusions

This paper proposes a design method for diffraction structure based on the dispersion of off-axis meta-lens by introducing a subwavelength grating splitting phase, which can achieve light splitting and focusing simultaneously. Meanwhile, the proposed structure with a diameter of 60 μm and a design wavelength of 1550 nm is designed and verified by simulation, and the imaging quality of the structure is analyzed. Simulation results show that the proposed structure can achieve higher resolution than the off-axis meta-lens at the same off-axis angle, and the proposed structure has a wider working band than the off-axis meta-lens with the same resolution, and the analysis shows that the imaging quality of the proposed structure is better than the off-axis meta-lens with the same parameters. The proposed structural design method and the analytical results have potential and beneficial applications in broad-band ultra-compact spectrometers.

## Figures and Tables

**Table 1:** Design parameters of double-layer diffraction structure.

Parameter	Value
Design wavelength	1550 nm
Numerical aperture of off-axis meta-lens	0.1
Phase distribution slope of subwavelength grating	4.562 × 10^5^
Off-axis Angle	45°
Focal length of off-axis meta-lens	5 mm

**Table 2:** Simulation parameters of the double-layer diffraction structure.

Parameter	Value
Design wavelength	1550 nm
Diameter of structure	60 μm
Off-axis Angle	10°
Focal length of off-axis meta-lens	137.08 μm
Phase distribution slope of subwavelength grating	11.203 × 10^4^
Unit structure period	500 nm

**Table 3:** Comparison table of performance metrics. (**a**) When having the same off-axis angle. (**b**) When having the same resolution.

(**a**)				
**Structure**	**Off-Axis Angle**		**Resolution**	**Strehl Ratio**
Off-axis meta-lens	10°		287.4 nm	3.09 × 10^−5^
Double-layer diffraction structure	10°		150.1 nm	0.76
(**b**)				
**Structure**	**Resolution**	**Off-Axis Angle**	**Operating Bandwidth**	**Strehl Ratio**
Off-axis meta-lens	150.1 nm	19.32°	1550~1701 nm	0.0052
Double-layer diffraction structure	150.1 nm	10°	1399~1852 nm	0.76

## Data Availability

The data supporting the plots and other findings of this study in this paper are available from the corresponding authors upon reasonable request.

## References

[1] Fattal D., Li J., Peng Z., Fiorentino M., Beausoleil R.G. (2010). Flat Dielectric Grating Reflectors with High Focusing Power. Nat. Photonics.

[2] Wang Y., Chen Q., Yang W. (2021). High-Efficiency Broadband Achromatic Metalens for near-IR Biological Imaging Window. Nat. Commun..

[3] Lin P., Lin Y., Lin J. (2021). Stretchable Metalens with Tunable Focal Length and Achromatic Characteristics. Results Phys..

[4] Shan D., Xu N., Gao J. (2022). Design of the All-Silicon Long-Wavelength Infrared Achromatic Metalens Based on Deep Silicon Etching. Opt. Express.

[5] Lin R., Wu Y., Fu B. (2021). Application of chromatic aberration control of metalens. Chin. Opt..

[6] Li M., Li S., Chin L.K. (2020). Dual-Layer Achromatic Metalens Design with an Effective Abbe Number. Opt. Express.

[7] Nalimov A.G., Kotlyar V.V., Stafeev S.S. (2023). A metalens-based optical polarization sensor. Comput. Opt..

[8] Shan D., Gao J., Xu N. (2022). Bandpass Filter Integrated Metalens Based on Electromagnetically Induced Transparency. Nanomaterials.

[9] Zuo R., Liu W., Cheng H. (2018). Breaking the diffraction limit with radially polarized light based on dielectric metalenses. Adv. Opt. Mater..

[10] Li Y., Cao L., Wen Z. (2019). Broadband quarter-wave birefringent meta-mirrors for generating sub-diffraction vector fields. Opt. Lett..

[11] Li R., Guo Z., Wang W. (2015). Arbitrary focusing lens by holographic metasurface. Photonics Res..

[12] Sajedian I., Lee H., Rho J. (2019). Double-Deep Q-Learning to Increase the Efficiency of Metasurface Holograms. Sci. Rep..

[13] Fu R., Li Z., Zheng G. (2021). Research development of amplitude-modulated metasurfaces and their functional devices. Chin. Opt..

[14] Avayu O., Almeida E., Prior Y. (2017). Composite Functional Metasurfaces for Multispectral Achromatic Optics. Nat. Commun..

[15] Jin J., Pu M., Wang Y. (2017). Multi-channel vortex beam generation by simultaneous amplitude and phase modulation with two-dimensional metamaterial. Adv. Mater. Technol..

[16] Wei Q., Huang L., Li X. (2017). Broadband multiplane holography based on plasmonic metasurface. Adv. Opt. Mater..

[17] Cheng H., Wei X., Yu P. (2017). Integrating polarization conversion and nearly perfect absorption with multifunctional metasurfaces. Appl. Phys. Lett..

[18] Bai W., Yang P., Wang S. (2019). Actively Tunable Metalens Array Based on Patterned Phase Change Materials. Appl. Sci..

[19] Yu P., Li J., Zhang S. (2018). Dynamic Janus Metasurfaces in the Visible Spectral Region. Nano Lett..

[20] She A., Zhang S., Shian S., Clarke D.R., Capasso F. (2018). Adaptive Metalenses with Simultaneous Electrical Control of Focal Length, Astigmatism, and Shift. Sci. Adv..

[21] Ren Z., Dong B., Qiao Q., Liu X., Liu J., Zhou G., Lee C. (2022). Subwavelength On chip Light Focusing with Bigradient All-dielectric Metamaterials for Dense Photonic Integration. InfoMat.

[22] Badri S.H., Gilarlue M.M. (2020). Coupling between Silicon Waveguide and Metal-Dielectric-Metal Plasmonic Waveguide with Lens-Funnel Structure. Plasmonics.

[23] Badri S.H., Gilarlue M.M., Gavgani S.G. (2020). Ultra-Thin Silicon-on-Insulator Waveguide Bend Based on Truncated Eaton Lens Implemented by Varying the Guiding Layer Thickness. Photonics Nanostructures Fundam. Appl..

[24] Khorasaninejad M., Chen W.T., Oh J. (2016). Super-Dispersive Off-Axis Meta-Lenses for Compact High Resolution Spectroscopy. Nano Lett..

[25] Zhu A.Y., Chen W.-T., Khorasaninejad M. (2017). Ultra-Compact Visible Chiral Spectrometer with Meta-Lenses. APL Photonics.

[26] Faraji-Dana M., Arbabi E., Arbabi A. (2018). Compact Folded Metasurface Spectrometer. Nat. Commun..

[27] Yang Z., Tang Y., Bayanheshig, Pan M., Cui J., Yang J. (2014). Optimization Design Method for Optical System of Prism-Grating Ultraspectral Imaging Spectrometers. Acta Opt. Sin..

[28] Yang Z., Albrow-Owen T., Cai W. (2021). Miniaturization of Optical Spectrometers. Science.

[29] Tang F., Ye X., Li Q. (2020). Dielectric Metalenses at Long-Wave Infrared Wavelengths: Multiplexing and Spectroscope. Results Phys..

[30] Liu W., Cheng H., Tian J. (2020). Diffractive Metalens: From Fundamentals, Practical Applications to Current Trends. Adv. Phys. X.

[31] Luo X. (2017). Sub-Wavelength Electromagnetism.

[32] Welford W.T. (1986). Aberrations of Optical Systems.

[33] Leng B., Chen M., Tsai D. (2023). Design, Fabrication, and Imaging of Meta-Devices. Acta Opt. Sin..

